# Mutations in valosin-containing protein (VCP) decrease ADP/ATP translocation across the mitochondrial membrane and impair energy metabolism in human neurons

**DOI:** 10.1074/jbc.M116.762898

**Published:** 2017-03-30

**Authors:** Marthe H. R. Ludtmann, Charles Arber, Fernando Bartolome, Macarena de Vicente, Elisavet Preza, Eva Carro, Henry Houlden, Sonia Gandhi, Selina Wray, Andrey Y. Abramov

**Affiliations:** From the ‡ Department of Molecular Neuroscience, UCL Institute of Neurology, London WC1N 3BG, United Kingdom,; the § Neurodegenerative Disorders Group, Research Institute Hospital 12 de Octubre (i+12), Madrid 28041, Spain,; the ‖ Institute of Neurology, MRC Centre for Neuromuscular Diseases, London WC1N 3BG, United Kingdom,; the ** Sobell Department of Motor Neuroscience and Movement Disorders, UCL Institute of Neurology, London WC1N 3BG, United Kingdom, and; the ¶ Biomedical Research Networking Center on Neurodegenerative Diseases (CIBERNED), Madrid 28041, Spain

**Keywords:** ADP, amyotrophic lateral sclerosis (ALS) (Lou Gehrig disease), ATP, induced pluripotent stem cell (iPS cell) (iPSC), mitochondria, ANT, VCP

## Abstract

Mutations in the gene encoding valosin-containing protein (VCP) lead to multisystem proteinopathies including frontotemporal dementia. We have previously shown that patient-derived *VCP* mutant fibroblasts exhibit lower mitochondrial membrane potential, uncoupled respiration, and reduced ATP levels. This study addresses the underlying basis for mitochondrial uncoupling using *VCP* knockdown neuroblastoma cell lines, induced pluripotent stem cells (iPSCs), and iPSC-derived cortical neurons from patients with pathogenic mutations in *VCP*. Using fluorescent live cell imaging and respiration analysis we demonstrate a *VCP* mutation/knockdown-induced dysregulation in the adenine nucleotide translocase, which results in a slower rate of ADP or ATP translocation across the mitochondrial membranes. This deregulation can explain the mitochondrial uncoupling and lower ATP levels in VCP mutation-bearing neurons via reduced ADP availability for ATP synthesis. This study provides evidence for a role of adenine nucleotide translocase in the mechanism underlying altered mitochondrial function in VCP-related degeneration, and this new insight may inform efforts to better understand and manage neurodegenerative disease and other proteinopathies.

## Introduction

Valosin-containing protein (VCP)^3^ (alias p97/TERA) is a ubiquitously expressed member of the type II AAA^+^ ATPase family. Mutations in *VCP* have been found in patients with inclusion body myopathy with early-onset Paget disease and frontotemporal dementia as well as familial amyotrophic lateral sclerosis (ALS). VCP has been linked to a variety of cellular functions including ubiquitin-proteasome system, protein degradation at the outer mitochondrial membrane, and autophagy but the underlying molecular mechanism in disease is yet to be fully understood.

Clues to the molecular mechanism associated with *VCP* mutations have come from model organisms that possess high inter-species homologies in the VCP protein. In particular, mutant mouse knock-in models mirror age-depandant motor-neuron dysfunction (1, 2). Abnormalities have been found in mitochondria cristae structures suggesting mitochondrial pathology in *VCP* mutation bearing cells (3). Our recent work has provided an insight into the observed mitochondrial pathologies through the utilization of a human dopaminergic neuroblastoma *VCP* knockdown cell line (SH-SY5Y), fibroblasts from patients carrying pathogenic mutations in the *VCP* gene and *VCP* knockdown mouse neurons, where we revealed an uncoupling of respiration from oxidative phosphorylation. Furthermore, it was shown that uncoupling leads to a lower mitochondrial membrane potential (indicator of cell health), higher respiration, and lower ATP levels in these cells (4). We hypothesized that the *VCP* mutation-induced uncoupling event may occur due to one of two reasons: 1) uncoupling proteins (UCPs) may alter the mitochondrial proton gradient, thus uncoupling respiration from oxidative phosphorylation. 2) Dysfunctional adenine nucleotide translocase (ANT1, SLC25A4), which is responsible for the simultaneous ADP import and ATP export across the mitochondrial inner membrane, leading to reduced ADP substrate for oxidative phosphorylation.

Other studies have investigated the effects of VCP on cellular health in patient fibroblasts, SH-SY5Y, as well as myoblasts (5) and this present study is the first to employ a disease-relevant neuronal model. In this current study, we set out to generate a human neuronal model of *VCP* mutations to explain the underlying mechanism of mitochondrial uncoupling in *VCP* mutant cells. To produce a human neuronal cell model, expressing mutant VCP at physiological levels, we generated iPSCs from patient fibroblasts and subsequently differentiated the cells to cortical neurons to study mitochondrial function. Live cell imaging techniques and a combination of *VCP* knockdown SH-SY5Y cell lines as well as iPSCs and iPSC-derived neurons enabled us to establish that a reduced activity of ANT1 leads to the uncoupling of mitochondria in *VCP* mutation bearing cells.

## Results

### Uncoupling of VCP mutation bearing cells is not caused by UCPs

**Figure 1.**
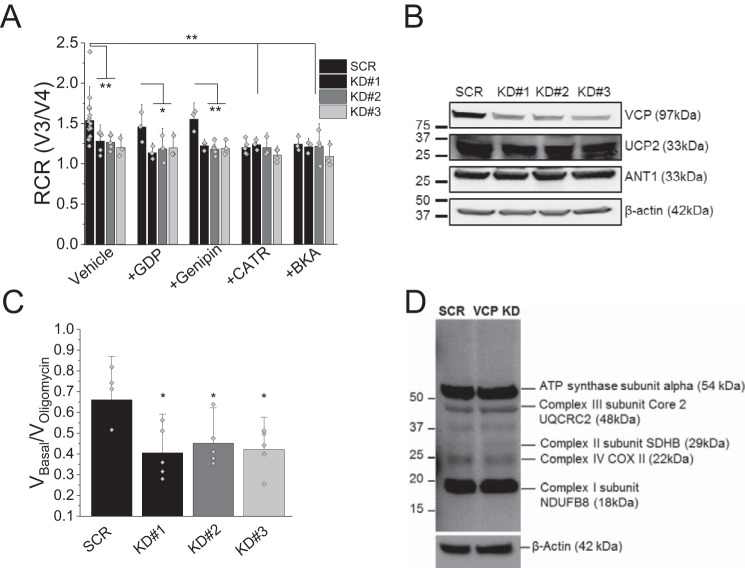
*A,* RCR is the ratio of state 3 respiration (ADP stimulated) to state 4 respiration (no ADP present), and is considered an indication of the degree of coupling of mitochondrial respiratory chain activity to oxidative phosphorylation. RCR of mitochondria in permeabilized SHY5Y control and *VCP* knockdown cells (KD#1, KD#2, KD#3) in the presence of vehicle control, uncoupling protein inhibitors (*GDP*; *Genipin*), or ANT inhibitors (*CATR*; *BKA*). *B,* SHY5Y SCR and KD cells were probed for VCP, ANT1, UCP2, and β-actin by Western blotting (cropped). *C,* quantification of *V*_Basal_/*V*_oligomycin_ in permeabilized SHY5Y control and VCP knockdown cells as an alternative indicator of coupling of mitochondrial respiratory chain activity to oxidative phosphorylation. *D,* full-length representative Western blot analysis of whole cell lysates from stable SCR SH-SY5Y cells and stable *VCP* knockdown SH-SY5Y cells probing for mitochondrial complexes and β-actin. *, *p* < 0.05; **, *p* < 0.01; ***, *p* < 0.001.

Our previous study provided evidence of lower ATP levels as well as uncoupled mitochondria (4). To investigate this further, we assessed mitochondrial coupling in stable *VCP* knockdown (KD) and scrambled (SCR) SH-SY5Y neuroblastoma cell lines. The *VCP* KD mimics the dominant-negative effect displayed by pathogenic loss-of-function *VCP* mutations. As previously shown by Bartolome *et al.* (4), *VCP* KD cells (clones KD#1, KD#2 and KD#3) displayed a significantly lower respiratory control rate (RCR; an indicator of coupling of oxidative phosphorylation and respiration) when compared with SCR cells (Fig. 1 A; *n* ≥ 3 experiments; *p* < 0.01). To assess whether the mitochondrial uncoupling in SH-SY5Y *VCP* KD cells is caused by UCPs, permeabilized cells were pre-exposed for 1 min to the UCP inhibitors GDP (100 μm) or Genipin (50 μm) and the coupling of oxidative phosphorylation and respiration was assessed (Fig. 1*A*). Application of GDP and Genipin did not alter the RCR of SCR or *VCP* KD cells indicating that UCPs are not the underlying reason for the mitochondrial uncoupling in VCP mutants (*n* ≥ 3 experiments). In support of this finding, Western blotting for UCP2 showed equal levels in control and *VCP* KD cells (Fig. 1*B*). Protein levels of mitochondrial electron transport chain (ETC) complexes were also found to be unchanged in *VCP* KD cells when compared with control suggesting that the mitochondrial phenotype is not caused by an alteration in ETC complex expression (Fig. 1*D*).

### Lack of ATP synthase substrate in VCP KD cells

Due to the difference in charge between ATP and ADP, changes in ANT activity can affect mitochondrial membrane potential and respiration. To test whether this is the underlying cause of the uncoupling event observed in *VCP* knockdown cells, we exposed SCR control and *VCP* KD cells to ANT inhibitors carboxyatractyloside (CATR; 5 μm) or bongkrekic acid (BKA) (5 μm) and RCR was assessed. Inhibition of ANT resulted in a significantly reduced RCR in SCR cells, which mirrors the RCR observed in SH-SY5Y *VCP* KD cells (Fig. 1*A*; *p* < 0.01; *n* ≥ 3 experiments). Furthermore, the lower RCR in the ANT inhibitor-treated SCR cell suggests that inhibition of ANT alone is able to uncouple mitochondria. There was no additional reduction in RCR in *VCP* KD cells exposed to ANT inhibitors suggesting that ANT is already inhibited and substrate (ADP) supply is reduced (Fig. 1*A*). To exclude a reduced ANT1 expression being the underlying reason, a Western blot analysis probing for ANT1 was undertaken. No changes in total ANT1 protein expression were found in *VCP* KD cells (Fig. 1*B*).

A proton leak through the ATP synthase could be the underlying reason for uncoupled mitochondria in *VCP* KD cells (6). Therefore, the irreversible ATP synthase inhibitor oligomycin (2 μg/ml) was applied (Fig. 1*C*). Oligomycin induced much smaller inhibition of respiration in *VCP* KD cells providing evidence that the uncoupling of these mitochondria is not caused by a proton leak in these cells. These results suggest that substrate supply in the form of ADP is limited in VCP KD cells leading to mitochondrial uncoupling.

### Generation of VCP mutant iPSCs and cortical neurons

To further our understanding of mutant-VCP disease mechanism, we employed a more physiologically relevant model in the form of patient-derived iPSCs and iPSC-derived neurons.

**Figure 2.**
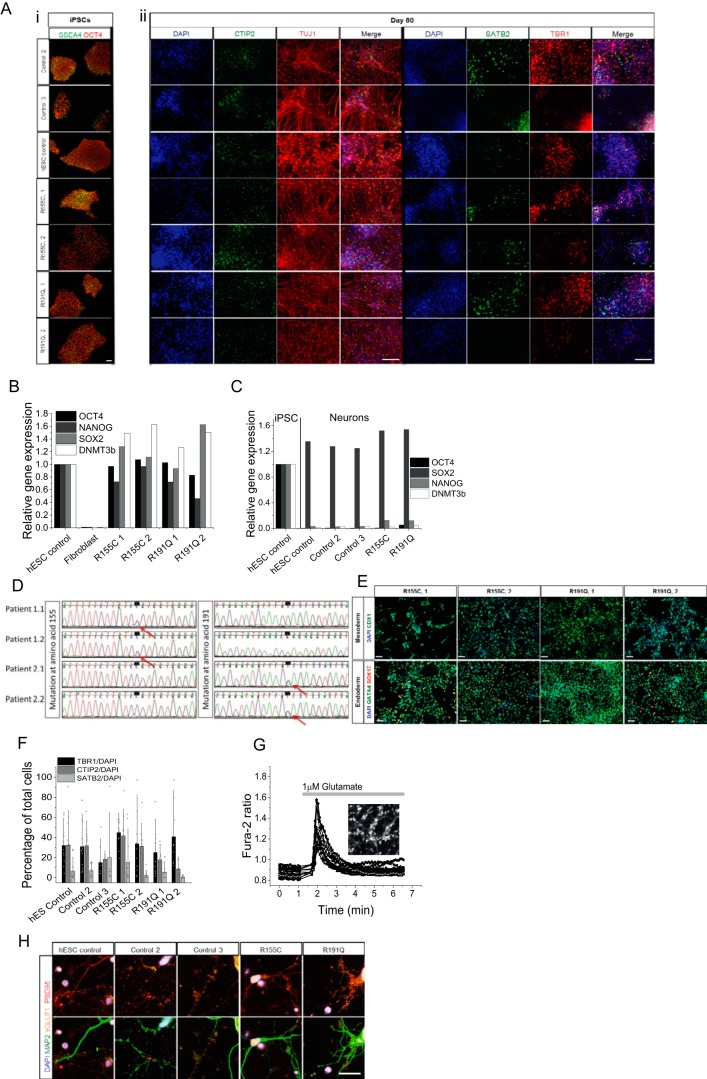
*A,* immunocytochemistry showing three control iPSCs and two patient-derived *VCP* mutant iPSCs lines (2 clones each). All stem cells show expression of pluripotency markers OCT4 and SSEA4 in stem cell conditions and have characteristic colony morphology. By day 80 of differentiation, β-III-tubulin positive neurons are formed expressing markers of deep (*TBR1*), middle (*CTIP2*), and upper (*SATB2*) cortical neurons. Size bar = 100 μm. *B,* qPCR analysis of four pluripotent stem cell markers in VCP patient-derived iPSCs and control fibroblasts, normalized to three housekeeping genes. *C,* qPCR analysis to represent faithful silencing of pluripotency genes in neuronal cultures, relative to hESC controls. *D,* DNA sequencing of iPSC-derived neuron genomic DNA showing mutated nucleotides corresponding to amino acid residues 155 and 191 (*arrows*). *E,* monolayer directed differentiation, showing the ability of newly derived iPSC lines to enter the endoderm and mesoderm differentiation pathways. *Size bar* = 50 μm. *F,* automated counting of mature neuronal markers displays no difference in the ability of control and VCP iPSCs to form deep (*TBR1*), middle (*CTIP2*), and upper (*SATB2*) layer cortical neurons. *G,* representative traces of Fura-2-loaded iPSC-derived neurons showing a transient cytosolic calcium response to physiological concentrations of glutamate. Representative image depicts iPSC-derived neurons loaded with Fura-2. *H,* iPSC-derived neurons express markers of glutamatergic neurons (*vGLUT1*) and functional synapses (*PSD95*). *Size bar* = 20 μm.

Fibroblasts from two patients carrying the R155C (most prevalent *VCP* mutation) and R191Q mutations were reprogrammed to iPSCs using episomal reprogramming factors (7). These patients both exhibited autosomal dominant inclusion body myopathy and frontotemporal dementia (FTD). The resultant iPSC clones exhibited similar expression of pluripotency markers to control hESCs at the protein (Fig. 2*A*) and RNA level (Fig. 2*B*). Reprogrammed cells exhibited a stable karyotype with unaltered g-banding (data not shown) and DNA sequencing confirmed that these stem cell lines retained the *VCP* mutation (Fig. 2*D*). The potential of the cells to differentiate into three germ layers was confirmed (Fig. 2*E*). Two clones from each patient were taken forward for further experiments and compared with two control iPSC lines and one control hESC line.

iPSCs were differentiated into cortical neurons, which are affected in VCP-linked FTD, as described previously (8). After 80 days of neurogenesis, control and *VCP* mutant lines generated similar proportions of deep (TBR1), middle (CTIP2), and upper (SATB2) cortical neurons (Fig. 2, *A*, *ii*, and *F*). The faithful silencing of pluripotency marker gene expression reinforces the footprint free reprogramming method (Fig. 2*C*). *SOX2* expression remains high; representing the neural stem cells that persist within the culture (9). The fact that *VCP* mutant iPSCs can generate cortical neurons at a similar efficiency to control lines hints at a normal capacity of *VCP* mutant cells for neurogenic differentiation and supports disease associated to aging rather than developmental abnormalities. The glutamatergic identity was confirmed by loading neurons with the cytosolic calcium indicator Fura-2 and application of a physiological concentration of glutamate (1 μm), which resulted in a transient intracellular calcium signal (Fig. 2*G*). Furthermore, a mature glutamatergic neuronal phenotype was validated via punctate vGLUT1 staining and expression of the post-synaptic marker PSD95 (Fig. 2*H*). Together, these results demonstrate an effective ability of the cells to differentiate into cortical neurons and represent an appropriate model to study *VCP* mutations in the cell type affected in FTD.

### VCP and ANT expression is not affected in patient-derived neurons

**Figure 3.**
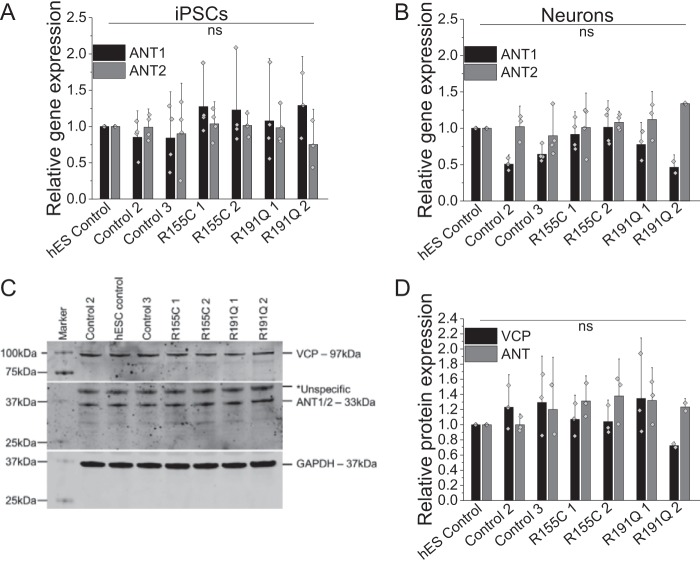
Quantification of *ANT1/2* gene expression in iPSC (*A*) and iPSC-derived neurons (*B*) by qPCR. Number of repeats are indicated in the bar chart. *C,* cropped Western blotting and *D,* densitometry analysis of VCP and ANT1/2 in iPSC-derived neurons. GAPDH was used as a loading control.

To further investigate the restricted ATP synthase substrate supply through ANT, we employed patient-derived iPSCs and cortical neurons bearing *VCP* mutations. First, we assessed *ANT1/2* gene expression on the mRNA level in iPSCs and iPSC-derived neurons where no significant changes could be found in R155C or R191Q mutation-bearing cells (Fig. 3, *A* and *B*). Protein expression levels of *VCP* and *ANT* in the iPSC-derived mutant neurons were also not changed when compared with control neurons (Fig. 3, *C* and *D*); reinforcing the dominant-negative theory that *VCP* mutations interfere with VCP hexamer function without affecting expression levels (10).

### Mitochondrial swelling in iPSC-derived neurons bearing a VCP mutation

**Figure 4.**
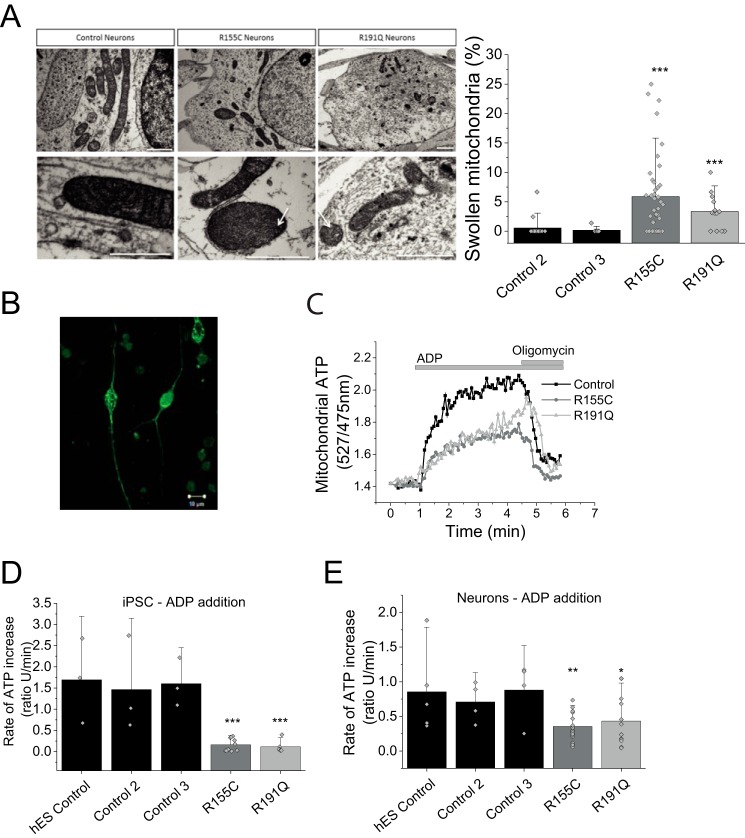
*A*, *i*, transmission electron microscopy images of mitochondria within control and mutant iPSC-derived neurons at day 80 of differentiation. Mutant neurons consistently display a small proportion of swollen, electron dense mitochondria. *Arrows* show damaged cristae. *Scale bar* represent 1 μm. *ii*, quantification of swollen mitochondria. *n* = 3 control, *n* = 3 R155C and *n* = 1 R191Q. *B,* representative image of a neuron transfected with mitAT1.03 probe. *C,* representative traces of mitochondrial ATP after ADP application. Oligomycin was applied as a negative control. *Scale bar* represent 10 μm. *D,* quantification of the rate of ATP increase in control and VCP mutation bearing iPS cells. *E,* quantification of the rate of ATP increase in control and VCP iPS-derived neurons. *, *p* < 0.05; *, *p* < 0.05; ***, *p* < 0.001.

Mitochondrial morphology was assessed in iPSC-derived neurons by EM (representative images in Fig. 4*A*, *i*). We observed that the number of swollen mitochondria in *VCP* mutant R115C neurons and R191Q was significantly higher (*p* < 0.001) when compared with control neurons (Fig. 4*A*, *ii*). This was confirmed in three independent neuronal inductions and not seen in three parallel control cultures. Additionally, swollen mitochondria displayed cristae that were disordered and interdigitation was disrupted (Fig 4*A, i*, *white arrows*). These observations in mitochondrial morphology further support that mitochondrial health is likely to be affected in *VCP* mutant iPSC-derived neurons. It should be noted that abnormal mitochondrial morphology has been witnessed in other *VCP* mutant models, such as R155H homozygous and heterozygous mice (3, 11).

### Lower ATP synthase activity in VCP mutation bearing cells

Oxygen consumption measurements are an indirect assessment of ATP synthase efficiency rather than ATP levels. To measure mitochondrial ATP levels in real-time, a FRET-based ATP probe was employed to allow assessment of ATP level changes (12). Patient-derived iPSCs and neurons were transfected with this probe where the fluorescent signal reflects mitochondrial ATP levels (Fig. 4*B*). The transfected cells were then permeabilized (40 μm digitonin to permeabilize the plasma membrane) in pseudo-intracellular solution and ATP synthase activity was assessed in the presence of mitochondrial substrates (5 mm malate and 5 mm glutamate) and ADP (500 nmol) (13). The applied ADP is translocated across the mitochondrial membranes via ANT where it is converted to ATP by the ATP synthase. ATP binds to the probe, which allows fluorescent live time recordings (Fig. 4*C*). All measurements were carried out in 3 control cell lines and two patient cell lines bearing either R155H or R191Q mutations (pool of two clones each) and statistical analysis was carried out on the pool of all control cell lines *versus* mutant cell lines.

iPSCs of affected patients had a significantly lower rate of ATP synthesis when compared with control (Fig. 4*D*). The rate of ATP increase in R155C (*n* = 10) and R191Q (*n* = 6) was significantly slower (*p* < 0.001) when compared with the pooled unaffected iPSC controls (*n* = 9; Fig. 4*D*). The rate was also significantly lower in neurons bearing the R155C mutation (*n* = 17; *p* < 0.001; Fig. 4*D*) and R191Q mutation (*n* = 12; *p* < 0.05) when compared with control iPSC-derived neurons (*n* = 13).

These results provide evidence that the ATP synthase in *VCP* mutant cells does not convert ADP to ATP as efficiently when compared with control cells. However, this approach does not distinguish between ATP synthase malfunction or a lower substrate (ADP) supply as the underlying basis for the difference in rate in ATP synthesis between control and *VCP* mutant cells.

### ADP/ATP translocation is inhibited in VCP mutation bearing cells

**Figure 5.**
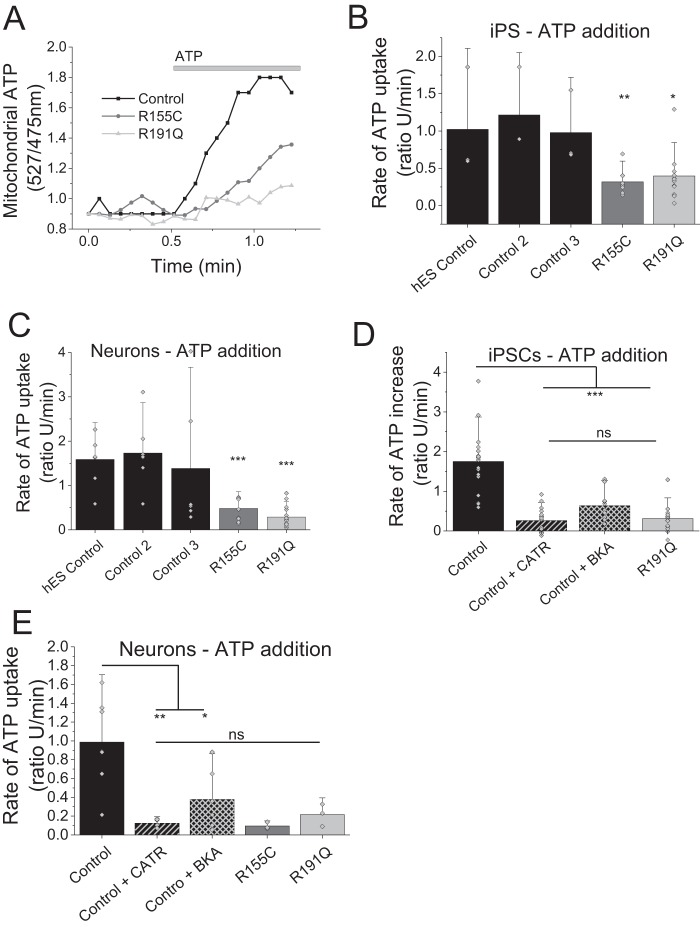
*A,* representative traces of mitochondrial ATP uptake. *B,* quantification of the rate of ATP uptake in control and VCP mutation bearing iPS cells. *C,* quantification of the rate of ATP uptake in control and VCP iPS-derived neurons. *D,* quantification of the rate of ATP uptake in control iPSCs or pre-exposed to ANT inhibitors (BKA/ATRAC) as well as mutation bearing iPSCs. *E,* quantification of the rate of ATP uptake in control neurons, neurons pre-exposed to ANT inhibitors (BKA/ATRAC), and mutations bearing neurons. *, *p* < 0.05; **, *p* < 0.01; ***, *p* < 0.001.

The translocase activity of ANT can be reversed through the application of high concentrations of ATP to permeabilized cells, which enables the import of ATP across the mitochondrial membranes. The translocated ATP is then bound by the mitochondrial ATP probe and the kinetic fluorescent measurements are a direct assessment of ATP translocation by ANT. It should be noted that this process is independent of the ATP synthase or ETC substrates and merely a measurement of ATP uptake, hence ANT activity (Fig. 5*A*). This method allowed investigation whether a lower substrate supply in the form of ADP is the reason for uncoupling.

Application of ATP (500 nmol) to permeabilized cells revealed a significantly slower ATP uptake into mitochondria of R155C iPSCs (*n* = 7; *p* < 0.001) as well as R191Q iPSCs (*n* = 14; *p* < 0.01) when compared with control cell lines (*n* = 8; Fig. 5, *A* and *B*). This observation was reproduced in iPSC neurons where ATP uptake into the mitochondria was significantly reduced in R155C neurons (*p* < 0.001) and R191Q when compared with control neurons (*n* = 8; Fig. 5*C*).

As a proof of principle, permeabilized control iPSCs were exposed to ANT inhibitors BKA or CATR for 30 min and the rate of ATP uptake was recorded (Fig. 5, *D* and *E*). Mitochondria pre-exposed to either ANT inhibitor displayed a significantly reduced ATP uptake (CATR *n* = 4; BKA *n* = 5; *p* < 0.001), mirroring the uptake observed in untreated patient iPSC (*n* = 8), when compared with control (*n* = 8). The rate of ATP uptake in iPSC-derived control neurons exposed to CATR (*p* < 0.01) and BKA (*p* < 0.05) was also significantly lower when compared with control neurons (*n* = 3; Fig. 5*E*). These results provide evidence that the mitochondrial uncoupling and lower ATP levels in *VCP* mutation bearing cells are caused by a lack of substrate (ADP) being translocated from the cytoplasm across the mitochondrial membranes.

## Discussion

We have previously shown that fibroblasts from patients harboring mutations in the *VCP* gene display mitochondrial uncoupling, seen by increased respiration, reduced oxidative phosphorylation, and lower ATP levels (4). In the present study we focused on the mechanism of this mitochondrial uncoupling. The UCP family and ANT both play an important role in regulating the coupling of the mitochondrial respiratory chain to oxidative phosphorylation. UCPs are mitochondrial carriers functioning via dissipation of the proton gradient across the inner membrane (14). To date, five human UCP homologues have been identified in different tissues and all are located at the mitochondria. UCP1 has a well characterized thermogenic role in brown adipose tissue. UCP3 is mainly expressed in both brown adipose tissue and muscle, whereas UCP2, UCP4, and UCP5 are mainly expressed in the central nervous system and therefore play key roles in neurons. Although only the role of UCP1 has been well characterized, it is generally accepted that all five UCPs uncouple oxidative phosphorylation from ATP synthesis by dissipating the proton gradient across the inner membrane (14).

Mitochondrial respiration analysis and Western blotting of *VCP* KD cells established that uncoupling of respiration from oxidative phosphorylation is not caused by UCPs or a proton leak through the ATP synthase. However, ANT inhibition in *VCP* KD did not lead to further uncoupling indicating that ANT could already be inhibited. Classical uncoupling is understood to be a reduction in coupling between the rate of electron transport in the respiratory chain (ADP independent; State 4) and ATP production (ADP-dependent; State 3) leading to a lower RCR. However, a reduced substrate supply (ADP) through limited uptake via ANT will lead to state 3 being inhibited resulting also in a lower RCR. Both scenarios decrease ATP levels and will ultimately lead to a lower bioenergetic status of the cell.

ANT1 is a mitochondrial protein that is thought to have an intrinsic uncoupling property (15–17). Additionally, ANT1 has been associated with the mitochondrial permeability transition pore and believed to be involved in mitochondria-mediated apoptosis (18). This study provided evidence that reduced ANT activity can lead to mitochondrial uncoupling as shown by ANT inhibition in SCR cells. The observed mitochondrial deficiency in *VCP* KD cells is in agreement with our previous work (4) where a lower NADH redox index and faster basal V2 respiration in *VCP* KD cells was reported. These features, together with a lower RCR, have been implicated in a number of neurodegenerative models. For example, Plun-Favreau *et al.* (6) showed similar phenotypes in an *HTRA2* knock-out mouse model where mutations in the *HTRA2* gene have been linked to Parkinson's disease. Additionally, studies have shown how the most common genetic cause of Parkinson's disease, G2019S mutations in the leucine-rich repeat kinase 2 gene (*LRRK2*), displayed uncoupling and low RCR implicating UCP2 and -4 proteins (19). Other transgenic mice for Huntington's disease reflected metabolic and thermoregulatory defects associated to uncoupling and reduced RCRs (20).

We extended our study by employing VCP patient-derived iPSCs and iPSC-derived neurons, which allowed us to investigate the dynamics of ADP and ATP translocation at the mitochondria in a human neuronal model. Investigations utilizing this highly energy-demanding cell type enabled us to validate previously observed mitochondrial abnormalities in the cell type affected by FTD.

Electron microscopy of mitochondria in iPSC-derived neurons displayed a significantly higher subset of swollen mitochondria that exhibit disordered inner mitochondrial membranes in VCP-deficient cells compared with those from control derived neurons. Damaged mitochondria were described as a common feature in VCP-associated disease models and in patient cell samples but there have been no reports showing this feature in neurons (5, 11, 21). For example, Johnson *et al.* (21) in 2015 showed damaged mitochondria in muscle cells from *Drosophila* and Nalbandian *et al.* (11) in 2012 showed mitochondrial megaconia and disrupted cristae in myoblasts from a *VCP* mouse model. The same group more recently showed damaged mitochondrial dynamics and function in fibroblasts and myoblasts from *VCP* patients (5). Here for the first time, we are showing EM images of damaged mitochondria in human neurons with *VCP* mutations. Additionally, the swollen mitochondria observed in the *VCP* mutant neurons could be an indicator of uncoupling, a feature originally described by Weinbach *et al.* (22) in 1963 and subsequently confirmed in a number of reports (23, 24).

The results suggest a decreased transport of ADP/ATP by ANT, which explains the mechanism behind the reduced ATP levels in the cells as previously shown in multiple other disease models (5, 25, 26). Inhibition of ADP/ATP transport could be observed in all cell types employed by this study. However, neurons require more energy than any other cell type (such as iPSC) and rely predominantly on oxidative phosphorylation for ATP production (with a very small impact of glycolysis). This makes neurons more vulnerable to mitochondrial uncoupling and inhibition of ATP transport (27).

Our data provide evidence that mutations in VCP lead to mitochondrial uncoupling due to a reduced ADP/ATP translocation by ANT. Here we provide unique analysis of the bioenergetics of *VCP* mutant human neurons with patient genotypes, allowing rational and relevant bioenergetic analysis in the cell type affected by neurodegeneration. The underlying deficiency in mitochondrial bioenergetics may help to explain disease in the context of *VCP* mutation and provide a focus for further studies into the brain metabolism in healthy aging and disease.

## Experimental procedures

### Cell culture

Human neuroblastoma (SH-SY5Y) cells were purchased from the European Collection of Cell Cultures (Health Protection Agency, Salisbury, UK) and maintained as previously described (28). Unless otherwise stated, SH-SY5Y cells were seeded at a density of 4 × 10^4^ cells/cm^2^ and maintained in Dulbecco's modified Eagle's medium (DMEM) supplemented with 10% (v/v) fetal bovine serum (FBS), 2 mm l-glutamine, and 1% (v/v) penicillin/streptomycin at 37 °C and 5% CO_2_. The scramble and VCP KD SH-SY5Y cells were generated by lentiviral infection with either a pool of 5 shRNA vectors or individuals shRNA (Thermo Fisher) as described previously by Bartolome *et al.* (4). Subsequent clonal selection was carried out using puromycin (1 μg/ml). Two different independent clones and a pool with 90% approximate reduction in gene expression were used in all experiments. 1 clone expressing nontargeting RNA vector was used as control (stable SCR).

### Cell differentiation

Fibroblast and iPSC cultures were performed as described previously (29, 30) and all reagents were obtained from Life Technologies, unless otherwise stated. Clinical data about the patients was described previously (4). Fibroblasts were cultured in DMEM with 10% fetal bovine serum and passaged with 0.05% trypsin. Reprogramming was performed as per the method detailed by Okita *et al.* (7) using episomal reprogramming plasmids (given as a gift from Shinya Yamanaka; Addgene plasmids #27077, #27078, and #27080) and the P2 nucleofection kit (Amaxa). One week after nucleofection, cells were passaged onto mouse embryonic feeders (AMS Bio) and media was changed to KO-DMEM, 20% knock-out serum replacement, 2 mm l-glutamine, non-essential amino acids, 50 μm β-mercaptoethanol, and 10 ng of FGF2 (Peprotech).

Newly formed iPSC colonies were manually picked and, together with control iPSCs and hESCs, were maintained in Essential 8 medium on Geltrex-coated plates. Karyotype analysis was performed by Cell Guidance Systems, Cambridge, UK. Differentiation to cortical neurons was performed as detailed (8, 34). 10 days of dual SMAD inhibition using 1 μm dorsomorphin and 10 μm SB431542 was followed by 70–90 additional days of differentiation in N2B27 media containing retinoids on laminin-coated dishes and coverslips. The hESC line SHEF6 was obtained from the United Kingdom Stem Cell Bank, control iPS line 1 was obtained from the lab of Dr. Tilo Kunath, and control iPS line 2 from Coriell repository.

For mesoderm and endoderm fate specification a defined monolayer protocol was followed, which allowed up-regulation of mesoderm- and endoderm-specific fate determinant gene networks, as published by others (31, 32). In brief, endoderm differentiation was achieved via a 3-day treatment consisting of 0.1 g/ml of activin (days 1–3), 80 ng/ml of FGF2 (days 1–3), 10 ng/ml of BMP4 (days 1–2), and 3 μm Chir (day 1). Mesoderm differentiation was directed via a 2-day treatment with 0.1 μg/ml of Activin, 20 ng/ml of FGF2, 10 ng/ml of BMP4, and 5 μm Chir.

### Immunocytochemistry

Cells were fixed in 4% paraformaldehyde for 15 min and washed three times in PBS containing 0.3% Triton X-100 (PBST), before blocking in 3% BSA in PBST for 20 min. Primary antibodies (Table 1) were incubated overnight at 4 °C in blocking solution, followed by three washes in PBST. Secondary antibodies were then added for 1 h at room temperature in the dark (Alexa Fluor, Invitrogen), DAPI was added for one wash at 1 μm and a final two washes was performed in PBST before mounting the coverslips for imaging on a Zeiss LSM microscope or a Zen confocal microscope. Cell counts were performed on 5 images per condition from three independent experiments, using the ITCN nuclear counting software plug-in for ImageJ with consistent settings between all conditions.

**Table 1:** **Antibodies used for immunocytochemistry and Western blotting**

Antibody name	Source	Dilution	Species
**OCT4**	Santa Cruz	1:500	gt
**SSEA4**	BioLegend	1:200	m
**MAP2**	Abcam	1:10,000	ch
**PSD95**	Abcam	1:1000	m
**vGLUT1**	Synaptic systems	1:1000	rb
**TUJ1**	Covance	1:1000	m/rb
**TBR1**	Abcam	1:500	rb
**SATB2**	Abcam	1:100	m
**CTIP**	Abcam	1:500	rat
**VCP**	Cell Signalling	1:1000	m
**ANT1/2/3**	Santa Cruz	1:200	gt
**UCP2 (C-20)**	Santa Cruz	1:200	gt
**GAPDH**	Ambion	1:5000	m
**β-actin**	Sigma	1:5000	rb
**hGATA-4**	Santa Cruz	1:100	m
**hSox17**	R&D	1:200	gt
**hCDX1**	Abcam	1:200	r
**OXPHOS complexes**	Abcam	1:250	m

### Western blotting analysis

Cells were lysed for 1 h at 4 °C in lysis buffer containing 10 mm Tris, pH 8, 140 mm NaCl, 1 mm EDTA, 0.5 mm EGTA, 1% Triton X-100, 0.1% sodium deoxycholate, 0.1% SDS plus protease and phosphatase inhibitors (Roche Applied Science), before centrifugation at 10,000 rpm for 15 min at 4 °C. Proteins were separated on NuPage 10% SDS-polyacrylamide gels (Novex) and transferred onto nitrocellulose membranes. Membranes were blocked in PBS containing 0.1% Tween and 3% milk solution. Primary antibodies were incubated in blocking solution overnight at 4 °C (Table 1). Subsequent washes were performed in PBS-Tween and secondary antibodies (800 nm Rockland and 680 nm Molecular Probes) were incubated for 1 h at room temperature. Final washes were in PBS and membranes were scanned on an Odyssey imaging system (Li-Cor). All gels have been run under the same experimental conditions.

### qPCR analysis

RNA was isolated from cells using TRIzol reagent and the manufacturer's instructions (Thermo Fisher). Reverse transcription was performed using SuperScript III Reverse Transcriptase and random hexamer primers (Thermo Fisher). qPCR was performed using Power SYBR Green master mix (Thermo Fisher) and the Agilent Mx300P qPCR system and software with annealing of primers at 60 °C (Table 2). All results are relative to the expression of three housekeeping genes, *GAPDH*,*CYCLOPHILIN*, and β-*ACTIN*.

**Table 2:** **Primers used for qPCR**

Gene	Forward Primer	Reverse Primer	Product
***GAPDH***	atgacatcaagaaggtggtg	cataccaggaaatgagcttg	177 bp
***CYCLOPHILIN***	ggcaaatgctggaccaaacac	ttcctggacccaaaacgctc	147 bp
**β-*ACTIN***	tcaccaccacggccgagcg	tctccttctgcatcctgtcg	351 bp
***OCT4***	ttctggcgccggttacagaacca	gacaacaatgaaaatcttcaggaga	218 bp
***SOX2***	catggcaatcaaaatgtcca	tttcacgtttgcaactgtcc	119 bp
***NANOG***	gcttgccttgctttgaagca	ttcttgactgggaccttgtc	256 bp
***ANT1***	aagatcttcaagtctgatgg	ataatatcggcccctttc	263 bp
***ANT2***	gcttgtcttgtatgatgaa	aatagttgccactgagaa	145 bp
***DNMT3b***	tttagggagaacgggaat	agcaccagtaagaagagt	88 bp

### Sanger sequencing

Genomic DNA was extracted from VCP cells using the QuickExtract DNA extraction solution (Epicenter) as per the manufacturer's instructions. The regions of the VCP mutations were PCR amplified with VCP exon 5 (forward, TTAAGACAGGTGGGGTGGAG; reverse, CCCATCTCAGTCTCCCAAAG) primers using POLYMERASE HERE (it may be: FastStart PCR Master (Roche Applied Science)) in PCR containing 10 μl of FastStart of PCR Master, 1 μl of each primer, 1 μl of DMSO, 10 μl of H_2_O, and 1 μl of extracted gDNA. PCR was performed using a 65td55 standard cycling program. PCR products were subsequently purified by incubation with Exonuclease I/FastAP^TM^ Thermosensitive Alkaline Phosphatase (Thermo Scientific) mixture as per the manufacturer's instructions. 5 μl of purified products were used in sequencing reactions with 0.5 μl of Big Dye (BigDye Terminator V.3.1 Cycle Sequencing Kit, Applied Biosystems), 2 μl of sequencing buffer (BigDye Terminator V.3.1 Cycle Sequencing Kit, Applied Biosystems), 1 μl of primer, and 2.5 μl of H_2_O. For the removal of BigDye Terminator, sequencing reactions were filtered through Sephadex (Sigma) containing filter plates (Corning). Sequencing was performed using an Applied Biosystems 3730XI Sequencer and analysis of results was carried out using the Sequencher software (Gene Codes Corporation).

### Transmission electron microscopy

Neurons were fixed in 3% gluteraldehyde (EM grade, Agar Scientific), 0.1 m sodium cacodylate, and 5 mm calcium chloride. Neurons were then post-fixed in osmium tetroxide, stained with uranyl acetate and Reynold's lead citrate, and dehydrated with stepwise washes with increasing concentrations of ethanol. Cells were then mounted in epoxy resin using propylene oxide to aid infiltration, which was hardened at 60 °C for 2 days. Ultrathin sections were taken using a diamond knife (Leica Reichert Ultracut), sections were mounted on copper mesh grids, stained with uranyl acetate, and images were taken using the Philips CM12 transmission electron microscope using the Olympus Soft Imaging System iTEM and MegaViewIII camera. Mitochondria were analyzed in triplicate from two control lines and one line from each patient. At least 15 fields of view were analyzed per line for every replicate.

### Oxygen consumption measurements

SH-SY5Y cells were permeabilized with 40 μm digitonin and subsequent re-suspension in a hypotonic medium containing 135 mm KCl, 10 mm NaCl, 20 mm HEPES, 0.5 mm KH_2_PO_4_, 1 mm MgCl_2_, 5 mm EGTA, and 1.86 mm CaCl_2_, pH 7.1. Oxygen consumption was measured in the Clark-type oxygen electrode (Hansatech) thermostatically maintained at 25 °C. Glutamate (5 mm), malate (5 mm), and inhibitors were indicated were added to the chamber before the measurement took place. To assess V3, 50 nmol of ADP was added and all data were obtained using the Oxygraph Plus system with chart recording software.

### Fura-2 imaging

Calcium measurements using fura-2 were generated using an epifluorescence inverted microscope equipped with a ×20 fluorite objective (33). Changes in cytosolic calcium levels were monitored in single cells using excitation light provided by a Xenon arc lamp, the beam passing monochromator at 340 and 380 nm (Cairn Research, UK). The emitted fluorescence light was reflected through a 515-nm long pass filter to a cooled CCD camera (Retiga, QImaging, Canada) and digitized to 12-bit resolution. All imaging data were collected and analyzed using software from Andor (Belfast, UK).

### Kinetic ATP measurements

To determine the ATP levels, patient-derived iPSC and neurons were transfected with a mitochondrial ATP probe (mitAT1.03), which was generated by Imamura *et al.* (12) using Effectine (Qiagen) according to the manufacturer's instructions. Confocal images were obtained using a Zeiss 710 VIS CLSM microscope equipped with a META detection system and a ×40 oil immersion objective. The FRET was quantified by the 527:475 nm ratio with an excitation of 405 nm and a filter from 515 to 580 nm. ATP kinetics were assessed in permeabilized iPSC and iPSC-derived neurons. To permeabilize the transfected cells, a buffer (0.137 m NaCl, 5 mm KCl, 0.7 mm, NaH_2_PO_4_, 25 mm, Tris-HCl, pH 7.1) containing a low concentration of digitonin (20 μm) was added to the cells. Upon permeabilization, the buffer containing digitonin was replaced by fresh buffer containing mitochondrial substrates (5 mm glutamate and 5 mm malate). Cells were allowed to rest for at least 5 min before measurements took place. Basal mitochondrial ATP levels were recorded for 1 min before 500 nmol of ADP/ATP was added to the imaging chamber (500 μl final volume). Oligomycin (2 μg/ml) was added as a negative control at the end of the ADP experiments.

### Statistical analysis

Statistical analysis and exponential curve fitting were performed using Origin 9 (Microcal Software Inc., Northampton, MA) software. Experimental data are shown as column scatter ± S.D. *n* represents experiments undertaken. Statistical analysis between samples was performed using a one-way analysis of variance with Bonferroni correction. Differences were considered to be significantly different if *p* < 0.05 (*).

### Ethics approval and consent to participate

Informed consent was obtained from all patients in this study. All experimental protocols were carried out according to approved guidelines and regulations by the National Hospital for Neurology and Neurosurgery and the Institute of Neurology joint research ethics committee (09/0272).

## Author contributions

M. H. R. L., C. A., F. B., M. D. V., and E. P. performed the experiments; M. H. R. L., C. A., and F. B. analyzed the data. A. Y. A., S. W., E. C., H. H. contributed reagents/materials/analysis tools. M. H. R. L., C. A., F. B., A. Y. A., S. W., S. G., and H. H wrote the paper. All authors read and approved the final manuscript.

## Footnotes

3 The abbreviations used are:

VCP: valosin-containing protein
ANT: adenine nucleotide translocase
UCPs: uncoupling proteins
KD: knockdown
ETC: electron transport chain
CATR: carboxyatractyloside
FTD: frontotemporal dementia
iPSC: induced pluripotent stem cell
SCR: scrambled
RCR: respiratory control rate
qPCR: quantitative PCR
BKA: bongkrekic acid.
